# Knowledge and attitudes toward emergency contraception among pharmacy students and community pharmacists in Poland

**DOI:** 10.3389/fpubh.2026.1763478

**Published:** 2026-02-13

**Authors:** Justyna Czekajewska, Dariusz Walkowiak, Anna Jelińska, Jan Domaradzki

**Affiliations:** 1 Department of Social Sciences and Humanities, Poznan University of Medical Sciences, Poznan, Poland; 2 Department of Organization and Management in Health Care, Poznan University of Medical Sciences, Poznan, Poland; 3 Department of Pharmaceutical Chemistry, Poznan University of Medical Sciences, Poznan, Poland

**Keywords:** attitudes, emergency contraception, ethics, knowledge, pharmacists, pharmacy students, Poland, reproductive health

## Abstract

**Background:**

Emergency contraception (EC) remains a contentious issue shaped by ethical, legal, and cultural factors. Pharmacists are often involved in counselling and dispensing EC, placing them at the intersection of ethical, legal, and professional responsibilities. This study examined the knowledge, attitudes, and predictors of EC-related opinions among community pharmacists and pharmacy students in Poland.

**Methods:**

A cross-sectional web-based survey was conducted in 2025 among 157 pharmacists and 223 pharmacy students. It assessed EC knowledge and attitudes. Data were analyzed using descriptive statistics, group comparisons, and logistic regression models to identify predictors of knowledge and attitudes.

**Results:**

Pharmacists scored higher on the Knowledge Index than students (*p* = 0.0035). Overall, 84.5% supported the use of EC, 92.6% opposed its legal prohibition, 40.2% favoured over-the-counter availability, and 40.8% supported minors’ access without parental consent. Logistic regression showed that younger age and contraceptive experience predicted greater support for EC use, while higher religiosity decreased it. Pharmacy students were more supportive than pharmacists of EC use and minors’ access without parental consent. Respondents with higher knowledge were more likely to support the inclusion of EC in national health policy and to acknowledge pharmacists’ educational role.

**Conclusion:**

While pharmacists demonstrated higher factual knowledge, students expressed more permissive attitudes toward EC access. Both groups showed knowledge gaps and uncertainty about legal frameworks. The findings highlight the need for clearer ethical and legal guidance in pharmacy education and practice concerning reproductive health services.

## Introduction

Postcoital contraception, commonly known as emergency contraception (EC), is a method of preventing pregnancy used after unprotected sexual intercourse or in cases where regular contraception has failed. It works primarily by preventing or delaying ovulation through the use of hormonal agents (1). EC is available in two forms: hormonal emergency contraceptive pills (ECPs) and copper intrauterine devices (IUDs). The effectiveness of EC depends on the active substance and the timing of administration. Levonorgestrel (LNG) can prevent up to 85% of pregnancies, while ulipristal acetate (UPA) can be effective in up to 97% of cases if taken before ovulation and within 24 h after unprotected intercourse (2). In Poland, since 2017, both LNG and UPA have been available by prescription (3). LNG may remain effective beyond the approved 72-h window, while UPA can be used for up to 120 h after intercourse (2, 4). Both methods share the same primary mechanism of action but are ineffective if ovulation has already occurred (2, 5).

Although studies show a high demand for modern birth control methods, according to the Contraception Policy Atlas 2025, Poland has had the lowest access to contraception in Europe for years (33.2%), far below the European average and rates observed in other countries in the region, including Hungary (40%) and Cyprus (42.1%). In contrast, access to contraception in Western Europe exceeds 90% (6). Experts have noted ongoing gaps in contraceptive counselling, particularly regarding methods, effectiveness, and availability. In Poland, the main barriers include limited access to gynaecologists, especially in smaller towns and rural areas; the prescription requirement for EC; insufficient sexual education; and persistent taboos around sexuality, reinforced by religion and conservative norms (7). Additionally, contraception is not reimbursed, and minors require guardian consent to obtain a prescription, which may further limit access for some groups, including adolescents (7).

Despite recent policy initiatives, public awareness and perceptions of EC in Poland remain limited. In 2024, the Polish Minister of Health introduced a pilot programme enabling pharmacists to provide selected reproductive health services, including consultations and the issuance of prescriptions for ulipristal acetate (UPA) (8). Under this initiative, pharmacists may conduct consultations and provide patient education, and, where appropriate, issue prescriptions (9). The programme remains in the pilot phase and is intended to evaluate the feasibility and outcomes of this approach before wider implementation.

Although awareness of emergency contraception in Poland is increasing, it remains limited, and misconceptions as well as unresolved concerns persist. A 2015 Centre for Public Opinion Research report found that 49% of respondents believed that EC was harmful to women’s health, 45% equated it with abortion, and 57% feared that easier access would encourage sexual promiscuity and irresponsibility in intimate relationships, while 56% believed it would promote earlier sexual initiation among youth (10). Such misconceptions are more common among individuals with lower levels of education, conservative political views, residents of smaller towns, and those who are highly religious (10). Left unchallenged, misinformation may reduce acceptance of EC and encourage unsafe alternatives. In response, reproductive health experts, including gynaecologists and pharmacists, are increasingly engaged in efforts to dispel myths and reduce the stigma surrounding EC.

Educational campaigns such as “Morning AFTER” (11), projects like “Contraception.PL” (12), and the NFZ pilot programme (8) have aimed to increase public awareness of EC and expand pharmacists’ involvement in patient consultations. According to the Ministry of Health, over 1,300 pharmacies have joined (13), and pharmacist-issued prescriptions for EC rose by more than 9% in the first quarter of 2025 compared with the last quarter of 2024 (14). Recent data indicate that EC is most commonly used by women aged 31–40 and 19–25 years (14). Public support for making EC available without prescription to individuals aged 15 and over has also risen by 7% (15).

While these data illustrate the recent outcomes of educational and advisory initiatives, there is still a lack of Polish representative studies among pharmacists and pharmacy students regarding their knowledge and attitudes towards EC, including their concerns about prescribing to minors without parental consent. There is also limited evidence on pharmacists’ perceptions of their educational role in this area. Meanwhile, previous studies conducted in various countries reveal that both pharmacy students and practising pharmacists demonstrate only partial and often inconsistent knowledge of EC. Although most are aware of EC and its clinical use, a detailed understanding of its mechanism of action and effectiveness is often limited, and misconceptions, such as confusing EC with abortion, remain widespread (16, 17). Studies with pharmacy professionals further suggest that while knowledge of dosing and side effects is generally adequate, awareness of the precise mechanisms and safety profile is weaker (18, 19). Among students, the picture is similarly mixed: only a minority achieves adequate knowledge, with gaps most evident around ulipristal acetate and the timing of levonorgestrel (20). Levels of knowledge are influenced by factors such as professional experience and educational exposure, with younger or less experienced practitioners typically performing worse (21, 22). Collectively, these findings indicate inconsistencies in EC-related knowledge, underscoring the importance of standardized education in pharmacy training and continuing professional development.

This study aims to fill this gap by comparing community pharmacists and pharmacy students in terms of: (1) their knowledge and opinions regarding EC, and (2) the factors influencing their attitudes toward EC. Understanding these differences may provide insight into how pharmacy education and professional training relate to practice realities and communication with patients, including minors, thereby informing future educational research in reproductive healthcare.

## Methods

### Study design

While this study formed part of a larger project examining the knowledge and attitudes of community pharmacists and pharmacy students towards ethical and legal dilemmas related to EC, it focuses on the assessment of the knowledge and opinions of the pharmaceutical community regarding EC and the factors influencing respondents’ attitudes.

Data were collected using an anonymous, web-based self-administered questionnaire completed by fourth- and fifth-year full-time pharmacy students at the Poznan University of Medical Sciences (PUMS) and by community pharmacists registered with the Wielkopolska Regional Chamber of Pharmacy in Poznan.

Following a comprehensive literature review, a survey instrument was developed to measure knowledge and attitudes towards emergency contraception and to identify the social and demographic factors shaping these perceptions (23).

### Ethical issues

The study was conducted in accordance with the principles of the Declaration of Helsinki (revised 2000) (24) and received approval from the Bioethics Committee of the Poznan University of Medical Sciences (approval no. KB-197/25, dated 19 March 2025). Written informed consent was obtained from all survey respondents before completing the survey.

### Participants and setting

The study population consisted of fourth- and fifth-year pharmacy students and practising pharmacists. The inclusion of these groups was based on two key considerations. First, by the end of the third year, pharmacy students at PUMS acquire foundational and preclinical knowledge and begin more specialised training. Fourth-year students learn to analyse patient cases, integrate knowledge from different fields, and interpret prescriptions, while also gaining greater exposure to practice. Fifth-year students typically complete their theoretical coursework and prepare for internships and the licensing examination, enabling them to analyse drug interactions and provide patient consultations more effectively. Second, in contrast to students, licensed community pharmacists possess full professional qualifications, practical experience, and legal authorisation to practise, and they are registered in the national register of pharmacists.

Participants included in the study had to meet specific criteria: (1) be pharmacy students at PUMS, or (2) practising pharmacists registered with the Wielkopolska Regional Chamber of Pharmacy in Poznan; (3) be fluent in Polish; (4) voluntarily consent to participate; and (5) provide written informed consent before completing the questionnaire.

### Research tools

A structured questionnaire was developed based on a literature review of EC contraception and in collaboration with experts in pharmacy, public health, medical sociology, and bioethics. Its design followed the guidelines of the European Statistical System (25). A pilot version was tested among 35 third-year pharmacy students, which resulted in the reformulation of two questions. The final questionnaire consisted of three sections. The first contained questions regarding the knowledge of EC. The second section included questions about respondents’ opinions and attitudes towards EC. The third part of the questionnaire included questions on respondents’ demographic characteristics.

All items were closed-ended with predefined response options. To ensure clarity, specialist terminology was avoided. Attitudes were assessed using a five-point Likert scale ranging from *Strongly disagree* to *Strongly agree*, with the option *I do not know* included for most questions.

A knowledge index was developed from a 12-item survey section to evaluate respondents’ understanding of EC and facilitate comparisons of knowledge across respondents. The items addressed both clinical aspects, such as active substances in EC and potential side effects, and regulatory issues, including EC access legality and pharmacists’ prescription authority. Correct answers earned positive points, unanswered items received zero points, and incorrect responses incurred negative points.

To assess religiosity, emphasizing the subjective impact of religious beliefs on daily life and community relationships, the validated Polish adaptation of the Religious Commitment Inventory-10 (RCI-10-PL) by Worthington et al. was used. In this study, the RCI-10-PL demonstrated high reliability (Cronbach’s *α* = 0.948, 95% CI: 0.938–0.957), and principal component analysis confirmed a satisfactory single-factor model fit.

### Data collection

Data were collected between March and July 2025 by one of the study participants (JC). Students were recruited during scheduled classes, and pharmacists were recruited during conferences and training sessions. Before completion, respondents were informed by a member of the principal investigator (JC) about the study’s purpose, voluntary nature, anonymity, confidentiality, and the absence of remuneration. They were also advised of their right to withdraw at any time without consequences.

After providing informed consent, respondents received a QR code which, when scanned with their smartphones, gave them access to the online questionnaire. Completion took approximately 10–15 min.

### Data analysis

Descriptive statistics were used to summarize the sociodemographic characteristics and responses to Likert scale questions, reported as frequencies and percentages of total responses. For variables with missing data, analyses were conducted using complete-case analysis; respondents with missing values for a given variable were excluded only from analyses involving that specific variable. Given the very low proportion of missing data (e.g., 0.3% for contraception use), no imputation procedures were applied.

Logistic regression analyses were employed to investigate the impact of sociodemographic variables on the factors influencing respondents’ attitudes toward EC, specifically their support for or opposition to distinct viewpoints. The models included available sociodemographic variables collected in the survey. Other potentially relevant confounders, such as socioeconomic status, prior formal education on EC, or professional work setting, were not included because these data were not collected.

All statistical analyses were performed using JASP (Version 0.19.3), with statistical significance defined at *p* < 0.05.

## Results

The sample was predominantly female (71.6%), with males accounting for 28.4% (Table 1). More than half of the respondents (58.7%) were students, while 41.3% were pharmacists. Half of the respondents lived in cities with over 500,000 inhabitants (50%), whereas 16.3% lived in small towns of up to 10,000 inhabitants; the remaining respondents were distributed across medium-sized localities.

**Table 1:** Participants characterises.

Characteristics	Total	
	(*n* = 380)	%
Gender		
Female	272	71.6
Male	108	28.4
Occupational status		
Student	223	58.7
Pharmacist	157	41.3
Domicile		
Up to 10,000 inhabitants	62	16.3
10–50,000 inhabitants	41	10.8
51–100,000 inhabitants	19	5
101–500,000 inhabitants	68	17.9
Above 500,000 inhabitants	190	50
Age (in years)		
Range	21–64	
Mean (95% CI)	30.2 (29.1–31.3)	
SD	10.8	
Median	24	
Religious commitment inventory-10		
Range	10–50	
Mean (95% CI)	22.7(21.6–23.8)	
SD	10.6	
Median	21.5	
Have you ever used any form of contraception?		
Yes	302	79.3
No	78	20.5
Missing	1	0.3

Respondents’ ages ranged from 21 to 64 years (M = 30.2 ± 10.8). Scores on the Religious Commitment Inventory-10 ranged from 10 to 50 (M = 22.7, 95% CI: 21.6–23.8; SD = 10.6; median = 21.5). The majority of respondents (79.3%) reported using contraception, whereas 20.5% reported not using any.

**Figure 1:**
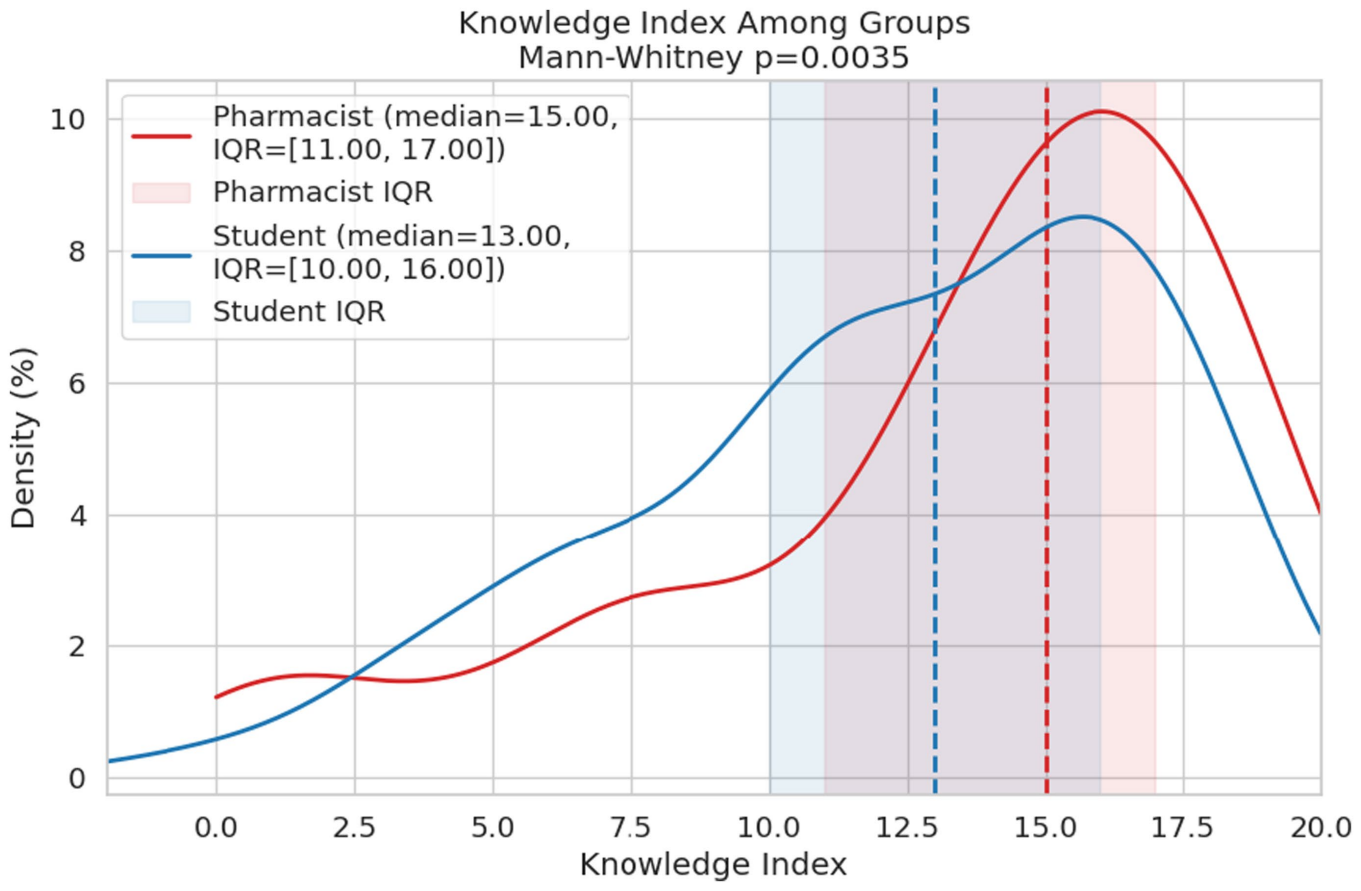
Knowledge index regarding emergency contraception of community pharmacists and pharmacy students.

Figure 1 shows that community pharmacists scored higher on the Knowledge Index (median = 15, IQR: 11–17) compared with pharmacy students (median = 13, IQR: 10–16), with the difference being statistically significant (Mann–Whitney *p* = 0.0035).

Table 2 summarizes the attitudes of community pharmacists and pharmacy students towards EC. The majority of respondents supported the use of EC (84.5% answered “rather yes” or “definitely yes”), while most opposed the idea of its legal prohibition (92.6% answered “definitely not” or “rather not”). Opinions were divided regarding over-the-counter availability, with 40.2% in favor and 52.9% against. Views on access for minors without parental consent were similarly split, with 40.8% supporting and 48.9% opposing. More than half of respondents (47.4%) agreed that EC should be reimbursed by the National Health Fund, while a large majority (83.9%) considered access to information about EC in Poland insufficient. Almost all respondents endorsed discussing EC in educational settings (95.8%) and involving pharmacists in related education (90%). Finally, 75.5% agreed that access to EC should form part of national health policy.

**Table 2:** Attitudes of community pharmacists and pharmacy students towards emergency contraception (EC).

Statement	Definitely not *n* (%)	Rather not *n* (%)	I do not know *n* (%)	Rather yes *n* (%)	Definitely yes *n* (%)
Supports the use of EC	11 (2.9)	27 (7.1)	21 (5.5)	108 (28.4)	213 (56.1)
EC should be legally prohibited	276 (72.6)	76 (20)	13 (3.4)	9 (2.4)	6 (1.6)
EC should be available without a prescription	71 (18.7)	130 (34.2)	26 (6.8)	81 (21.3)	72 (18.9)
A minor should be able to obtain a prescription for EC without parental consent	83 (21.8)	103 (27.1)	39 (10.3)	97 (25.5)	58 (15.3)
EC should be reimbursed by the National Health Fund	59 (15.5)	90 (23.7)	51 (13.4)	100 (26.3)	80 (21.1)
Access to information about EC in Poland is sufficient	138 (36.3)	181 (47.6)	25 (6.6)	30 (7.9)	6 (1.6)
The use of EC should be discussed within education	4 (1.1)	4 (1.1)	8 (2.1)	92 (24.2)	272 (71.6)
Access to EC should be part of the national health policy	16 (4.2)	21 (5.5)	56 (14.7)	124 (32.6)	163 (42.9)
Pharmacists should be involved in education about EC	4 (1.1)	11 (2.9)	23 (6.1)	123 (32.4)	219 (57.6)

Table 3 presents the results of logistic regression analyses evaluating community pharmacists’ and pharmacy students’ attitudes toward emergency contraception (EC), focusing on four outcomes: support for EC use, belief that EC should be legally prohibited, support for EC availability without a prescription, and support for minors obtaining EC without parental consent.

**Table 3:** Logistic regression parameters for community pharmacists’ and pharmacy students’ attitudes towards emergency contraception (EC): Part 1.

Regression parameters	Supports the use of EC	EC should be legally prohibited	EC should be available without a prescription	A minor should be able to obtain a prescription for EC without parental consent
	OR (95% CI)	OR (95% CI)	OR (95% CI)	OR (95% CI)
Intercept	108.856*** (18.831; 629.272)	0.001*** (0.000; 0.017)	11.434*** (4.895; 26.708)	0.495 (0.114; 2.146)
Age				1.066*** (1.029; 1.104)
Indeks wiedzy				
RCI-10	0.865*** (0.827; 0.904)	1.152*** (1.082; 1.226)	0.916*** (0.893; 0.940)	0.909*** (0.885; 0.933)
Men vs. women				
Students vs. pharmacists	2.869* (1.134; 7.257)			3.282** (1.523; 7.074)
Individuals who have used contraception vs. Individuals who have never used contraception	4.586** (1.764; 11.923)	0.236* (0.069; 0.814)		
Residents of municipalities with 10,000–50,000 inhabitants vs. those with up to 10,000 inhabitants			0.281** (0.111; 0.717)	
Residents of municipalities with 50,000–100,000 inhabitants vs. those with up to 10,000 inhabitants				
Residents of municipalities with 100,000–500,000 inhabitants vs. those with up to 10,000 inhabitants				
Residents of municipalities with over 500,000 inhabitants vs. those with up to 10,000 inhabitants			0.374** (0.189; 0.739)	
*R*^2^ Nagelkerke	0.468	0.400	0.223	0.223
*p*-value for model	0.022	0.021	0.038	0.002

Significant predictors include age (OR = 1.066, 95% CI: 1.029–1.104, *p* < 0.001) for supporting EC use, and the RCI-10 index, which is significant across all models (e.g., OR = 0.865, 95% CI: 0.827–0.904, *p* < 0.001 for EC support; OR = 1.152, 95% CI: 1.082–1.226, *p* < 0.001 for EC prohibition; OR = 0.916, 95% CI: 0.893–0.940, *p* < 0.001 for over-the-counter availability; OR = 0.909, 95% CI: 0.885–0.933, *p* < 0.001 for minors’ access).

Pharmacy students show greater support than pharmacists for EC use (OR = 2.869, 95% CI: 1.134–7.257, *p* < 0.05) and for minors’ access to EC without parental consent (OR = 3.282, 95% CI: 1.523–7.074, *p* < 0.01). Individuals with personal contraception experience are more likely to support EC use (OR = 4.586, 95% CI: 1.764–11.923, *p* < 0.01) and less likely to support its prohibition (OR = 0.236, 95% CI: 0.069–0.814, *p* < 0.05).

Respondents residing in municipalities with 10,000–50,000 inhabitants (OR = 0.281, 95% CI: 0.111–0.717, *p* < 0.01) or more than 500,000 inhabitants (OR = 0.374, 95% CI: 0.189–0.739, *p* < 0.01) are less likely to support EC use compared with those living in municipalities of ≤10,000 residents. Nagelkerke *R*^2^ values range from 0.223 to 0.468, with the highest explanatory power observed for EC support (*R*^2^ = 0.468), and overall model *p*-values (0.002–0.038) indicate statistical significance.

Table 4 presents the results of logistic regression analyses evaluating community pharmacists’ and pharmacy students’ attitudes toward emergency contraception (EC), focusing on five outcomes: (1) support for EC reimbursement by the National Health Fund, (2) perception of sufficient access to EC information in Poland, (3) belief that EC use should be discussed within education, (4) support for including EC access in national health policy, and (5) support for pharmacists’ involvement in EC education.

**Table 4:** Logistic regression parameters for community pharmacists’ and pharmacy students’ attitudes towards emergency contraception (EC): Part 2.

Regression parameters	EC should be reimbursed by the National Health Fund	Access to information about EC in Poland is sufficient	The use of EC should be discussed within education	Access to EC should be part of the national health policy	Pharmacists should be involved in education about EC
	OR (95% CI)	OR (95% CI)	OR (95% CI)	OR (95% CI)	OR (95% CI)
Intercept	2.615*** (1.544; 4.429)	0.071*** (0.028; 0.179)	76.406*** (5.930; 984.537)	43.669*** (11.916; 160.027)	1.025 (0.201; 5.230)
Age					
Knowledge index			1.227** (1.057; 1.425)		1.191** (1.061; 1.338)
RCI-10	0.967** (0.948; 0.987)	1.037* (1.006; 1.070)	0.915** (0.859; 0.975)	0.904*** (0.872; 0.938)	
Men vs. women					
Students vs. pharmacists		0.424* (0.208; 0.865)			10.484*** (2.646; 41.554)
Individuals who have used contraception vs. individuals who have never used contraception				4.541*** (2.029; 10.162)	
Residents of municipalities with 10,000–50,000 inhabitants vs. those with up to 10,000 inhabitants					
Residents of municipalities with 50,000–100,000 inhabitants vs. those with up to 10,000 inhabitants					
Residents of municipalities with 100,000–500,000 inhabitants vs. those with up to 10,000 inhabitants					
Residents of municipalities with over 500,000 inhabitants vs. those with up to 10,000 inhabitants					
*R*^2^ Nagelkerke	0.042	0.067	0.265	0.340	0.146
*p*-value for model	0.001	0.020	0.004	<0.001	0.004

The table reports odds ratios (OR) with 95% confidence intervals (CI), along with Nagelkerke *R*^2^ and model *p*-values to assess model fit. Significant predictors include the RCI-10 index, which remains significant across most models (e.g., OR = 0.967, 95% CI: 0.948–0.987, *p* < 0.01 for reimbursement; OR = 1.037, 95% CI: 1.006–1.070, *p* < 0.05 for information access; OR = 0.915, 95% CI: 0.859–0.975, *p* < 0.01 for education discussion; OR = 0.904, 95% CI: 0.872–0.938, *p* < 0.001 for national health policy).

The Knowledge Index is also a significant predictor for EC inclusion in national health policy (OR = 1.227, 95% CI: 1.057–1.425, *p* < 0.01) and pharmacists’ involvement in EC education (OR = 1.191, 95% CI: 1.061–1.338, *p* < 0.01). Pharmacy students are less likely than pharmacists to believe that access to EC information in Poland is sufficient (OR = 0.424, 95% CI: 0.208–0.865, *p* < 0.05), yet more likely to support pharmacists’ involvement in EC education (OR = 10.484, 95% CI: 2.646–41.554, *p* < 0.001). Individuals with personal contraception experience are more likely to support the inclusion of EC in national health policy (OR = 4.541, 95% CI: 2.029–10.162, *p* < 0.001). No significant effects were observed for municipality size, age, or gender across any of the models. Nagelkerke *R*^2^ values range from 0.042 to 0.340, with the highest explanatory power observed for EC inclusion in national health policy (*R*^2^ = 0.340). Overall model *p*-values (<0.001 to 0.020) indicate statistical significance across all models.

## Discussion

The results of this study reveal notable differences in attitudes toward EC between community pharmacists and pharmacy students. This variation may reflect differences in knowledge levels, professional experience, and social context within these groups. While pharmacists achieved higher scores on the knowledge index, students expressed more permissive views regarding EC use and its availability to minors without parental consent. These findings suggest that higher factual knowledge does not necessarily translate into more supportive attitudes toward EC access, highlighting the complex relationship between knowledge, ethical reasoning, and professional decision-making. Similar inconsistencies between pharmacists’ knowledge, attitudes, and professional practice have also been reported in other settings, particularly in relation to ethical standards and codes of professional conduct (26).

The discrepancies observed in this study are consistent with previous research on EC. In a U.S. study conducted among pharmacy students, researchers reported inconsistencies between knowledge, attitudes, and dispensing practices: while 32% of respondents admitted limited understanding of EC’s mechanism of action and only 26.7% felt competent to provide counselling, 42.8% declared willingness to dispense EC (27). These findings indicate that supportive attitudes toward EC access do not necessarily correspond with adequate theoretical knowledge or counselling competence, underscoring the role of both cognitive and contextual factors. Similarly, a Polish study found that only 51.6% of pharmacy personnel provided patients with at least basic information about EC, with counselling quality varying by education level and workplace setting. Substantial knowledge gaps were also identified: 63% of respondents lacked full knowledge of all EC methods, and only 43% correctly recognized that EC is not abortifacient (28). Insufficient knowledge regarding emergency contraception has been documented across multiple international settings. Studies conducted in Nigeria (21), Nepal (19), Jamaica (29), and South Africa (30) indicate persistent misconceptions among healthcare professionals, including beliefs that EC may act as an abortifacient, harm fetal development, or pose significant health risks when used repeatedly. Taken together, these findings suggest that pharmacists’ knowledge, similar to that of other healthcare professionals, often remains partial. Persistent gaps regarding EC mechanisms, available methods, and safety profiles may adversely affect the quality of patient counselling and influence individuals’ decisions about EC use.

A second key conclusion concerns the regulatory framework governing EC and the implementation of pharmacy-based services. While the vast majority of respondents opposed a legal ban on EC, opinions remained divided regarding over-the-counter availability and minors’ access without parental consent, indicating persistent regulatory and ethical uncertainty. Pharmacists’ more cautious attitudes, despite higher knowledge levels, may reflect greater awareness of legal responsibilities and the potential professional consequences of EC provision.

Previous studies conducted in Poland indicate that, although many pharmacists oppose refusing to dispense emergency contraception, the expansion of over-the-counter availability remains controversial (31–33). Merks et al. identified two principal sources of this controversy. First, ethical concerns play a central role, particularly differing interpretations of the beginning of human life. It should therefore be emphasized that emergency contraception, including ulipristal acetate and levonorgestrel, is not abortifacient and does not prevent implantation, as its mechanism of action consists of preventing or delaying ovulation (33–36). Second, legal considerations further contribute to the debate, particularly uncertainty surrounding the conscience clause, which allows healthcare professionals to refuse procedures that conflict with their moral or religious beliefs (33, 37). Studies suggest that many healthcare professionals perceive existing legislation as insufficiently clear regarding the scope and application of this clause, including its potential relevance to pharmacists (31).

These findings should also be interpreted in light of broader cultural and systemic determinants. Previous Polish research indicates that religiosity and moral norms rooted in Catholic teaching significantly shape healthcare professionals’ attitudes toward ethically sensitive services, including reproductive health care and conscientious clause (37–39). Moreover, studies conducted in broader European and international contexts demonstrate that religion, conscience-based reasoning, and institutional arrangements within healthcare systems play a critical role in shaping professionals’ responses to controversial clinical practices, particularly in areas related to reproductive health (40–42). Ambiguity surrounding the legal scope of the conscience clause and its uneven institutional implementation has been shown to generate ethical tension and uncertainty among medical professionals, further influencing their decision-making in practice (38, 43).

However, in Poland, the conscience clause does not formally apply to pharmacists. Any unauthorized invocation of this regulation may therefore restrict patients’ access to guaranteed healthcare services and expose pharmacists to legal consequences, particularly given the limited window of effectiveness of emergency contraception (33).

The third key conclusion of this study concerns predictors influencing attitudes toward EC. Age, contraceptive experience, and place of residence emerged as significant factors, with younger respondents, individuals reporting contraceptive use, and residents of smaller towns expressing more supportive views toward EC availability, including over-the-counter access and access for minors without parental consent.

The over-the-counter availability of emergency contraception has long been a contentious issue in Poland. CBOS surveys indicate that public opinion has remained divided over time. In 2015, attitudes toward over-the-counter access were nearly evenly split, with comparable proportions of respondents supporting and opposing this policy, often expressing concern about unrestricted access for younger individuals (10). A follow-up survey conducted in 2024 showed a modest shift toward greater acceptance, with approximately half of respondents supporting over-the-counter availability of the “morning-after pill,” while a substantial proportion remained opposed (15). These findings suggest persistent social divisions in attitudes toward EC access, shaped by age, place of residence, education level, and broader worldview (10, 15).

The present study reflects these patterns while also indicating a growing openness toward over-the-counter EC among residents of smaller towns. Nevertheless, EC remains less available in rural pharmacies than in urban areas, pointing to ongoing disparities in service distribution (44, 45). Structural inequalities, including lower income levels, higher rates of underinsurance, and reduced access to specialist healthcare in rural areas, may further limit access to reproductive health services and reliable contraceptive information (7, 44, 45).

Beyond legal availability, access to information emerged as a key determinant of EC use. Evidence from several European countries, including Italy, Germany, the United Kingdom, France, and Spain, highlights the role of stigmatization, insufficient counselling, and communication barriers in shaping women’s experiences with EC and their healthcare decisions (46). Consistent with these findings, respondents in the present study perceived access to EC-related information as insufficient, while higher knowledge levels were associated with stronger support for including EC in national health policy and greater recognition of pharmacists’ educational role.

The final key conclusion concerns professional attitudes toward patient support and education. Pharmacy students more frequently emphasized the importance of nonjudgmental counselling and public education, whereas practising pharmacists’ attitudes appeared more constrained by ethical concerns, stereotypes, or perceived legal risks. Similar barriers have been documented in studies conducted in Nigeria, the United States, and Australia, where pharmacists’ personal beliefs and institutional practices influenced EC provision (21, 47–50).

A meta-analysis further indicates that willingness to dispense EC to men, partners, or other third parties remains limited across settings (5).

Overall, national and international evidence indicate that the provision of EC, in contexts where it is offered, requires adequate preparation of both pharmacy staff and facilities. Implementing extended-access services, such as pharmacist dispensing, would require improving pharmacists’ and students’ knowledge of EC availability, mechanisms of action, and relevant legal and ethical frameworks. Education in this area constitutes an important aspect of pharmacists’ professional competence, as many patients demonstrate limited knowledge about reproductive health and contraception.

Research from other contexts has examined the association between EC availability, pharmacist counselling, and outcomes such as reduced rates of unintended pregnancies and related health complications (51–53). Clear professional guidelines for dispensing EC may contribute to greater consistency and neutrality in patient interactions. Ultimately, strengthening pharmacists’ educational preparation and understanding of ethical and cultural contexts may contribute to more consistent reproductive healthcare practices.

### Limitations

This study has several limitations that should be considered when interpreting its findings. First, the research employed a cross-sectional design, which captures attitudes and knowledge at a single point in time and therefore does not allow for causal inference. Longitudinal designs could provide deeper insight into how pharmacists’ and students’ attitudes toward EC evolve in response to experience, policy changes, or educational reforms. Second, convenience sampling was used, involving pharmacy students from a single university and pharmacists registered in one regional chamber, which may limit the generalizability of the findings to all pharmacists in Poland. Future studies should include larger, nationally representative samples to improve external validity. Third, the use of a self-administered web-based questionnaire may have introduced response bias related to social desirability or self-selection, as individuals with stronger opinions about EC may have been more likely to participate. Fourth, although the questionnaire demonstrated strong internal reliability, it relied on self-reported data, which may not fully reflect actual professional behaviors, such as dispensing practices or the quality of patient counselling. In addition, several potentially relevant confounding variables, such as respondents’ socioeconomic status, prior formal education or training related to emergency contraception, and pharmacists’ specific work setting, were not collected. Consequently, these factors could not be adjusted for in the statistical analyses and may have influenced the observed associations. Fifth, although the questionnaire was reviewed by subject-matter experts and pilot-tested, it did not undergo formal psychometric validation, which may affect the precision of some measured constructs. Consequently, a degree of measurement bias cannot be excluded. Subsequent studies should include full validation procedures to strengthen the methodological robustness of the instrument. The study sample was predominantly female, which may limit the generalizability of the findings to male pharmacists and students. However, this distribution reflects the gender structure of the Polish pharmacy workforce and pharmacy education system rather than a sampling imbalance. Sixth, the study did not directly assess institutional or cultural determinants, such as workplace policies, religious affiliation, or formal exposure to EC-related education during training, which may further explain observed differences in attitudes. Qualitative or mixed-method approaches, including interviews or focus groups, could help elucidate the ethical reasoning and contextual factors underlying pharmacists’ and students’ views on EC. Finally, cultural and systemic factors specific to Poland, including the moral influence of religion, the legal framework surrounding the conscience clause, and the organization of the healthcare system, may have shaped respondents’ attitudes toward EC. Accordingly, the findings should be interpreted within the national context, and future comparative studies should consider cross-country differences in cultural and regulatory environments.

Despite these limitations, this study also has important strengths. It is one of the first to examine pharmacists’ and pharmacy students’ knowledge and attitudes toward emergency contraception in Poland within an explicit ethical and legal framework. By integrating quantitative data on knowledge, religiosity, and demographic variables, the study provides a comprehensive overview of factors shaping attitudes toward EC access and use. The findings offer valuable preliminary insights into how current and future pharmacists perceive their educational and counselling roles in reproductive healthcare and may inform curriculum development, professional training, and public health initiatives aimed at improving equitable access to EC. Overall, this study contributes to broader international discussions on pharmacists’ ethical responsibilities and the expansion of pharmacy-based reproductive health services.

## Conclusion

This study revealed significant differences in knowledge and attitudes toward EC between pharmacists and pharmacy students. While pharmacists demonstrated higher factual knowledge, students expressed more supportive views regarding EC use and minors’ access without parental consent. Both groups exhibited knowledge gaps and uncertainty regarding legal regulations, indicating that professional experience alone does not ensure consistent understanding or attitudes. Age, religiosity, contraceptive experience, and place of residence emerged as key predictors shaping respondents’ views, highlighting the combined influence of cognitive and contextual factors on professional reasoning.

Although the majority of respondents opposed legal restrictions on EC, opinions remained divided regarding over-the-counter availability and minors’ independent access. These findings reflect persistent ethical, cultural, and regulatory tensions surrounding EC in Poland and underscore the need for clearer professional guidance and targeted educational interventions. Improving pharmacists’ knowledge and ethical competence may support more informed and consistent decision-making in pharmacy practice.

Based on these findings, several recommendations can be proposed to inform educational, professional, and policy initiatives aimed at strengthening pharmacists’ roles in reproductive healthcare:

1. Integrate structured education on reproductive health and ethical decision-making into undergraduate pharmacy curricula, with emphasis on pharmacological knowledge, patient communication, and relevant legal regulations, including the conscience clause.
2. Expand continuing professional development programs to include ethical reasoning, counselling skills, and case-based discussions addressing moral and legal dilemmas in reproductive health services.
3. Develop clear national professional guidelines defining pharmacists’ roles, rights, and responsibilities in providing or declining EC-related services, ensuring lawful, transparent, and patient-centred practice.
4. Strengthen interprofessional collaboration and ethical dialogue among pharmacists, physicians, and educators to promote shared understanding of legal obligations and professional values in reproductive healthcare.
5. Improve access to clear, evidence-based information for both pharmacists and the public on the ethical, legal, and professional aspects of EC to support informed decision-making.
6. Address inequalities in pharmacy service provision, particularly in rural areas, by ensuring equitable access to competent counselling and professional support regardless of patients’ place of residence or worldview.

## Data Availability

The original contributions presented in the study are included in the article/supplementary material, further inquiries can be directed to the corresponding author.
